# A conceptual framework for clinical and translational virtual community engagement research

**DOI:** 10.1017/cts.2022.479

**Published:** 2022-10-28

**Authors:** Michael Rubyan, M. Grace Trinidad, Kerry A. Ryan, Meghan Spiroff, Susan Goold, Jade Burns, Karen Calhoun, Zachary Rowe, Ayşe G. Büyüktür, Patricia Piechowski, Jodyn Platt

**Affiliations:** 1 School of Public Health, University of Michigan, Ann Arbor, MI, USA; 2 Institute for Healthcare Policy & Innovation, University of Michigan, Ann Arbor, MI, USA; 3 National Hemophilia Program Coordinating Center, American Thrombosis & Hemostasis Network, Rochester, NY, USA; 4 Center for Bioethics and Social Sciences in Medicine, University of Michigan, Ann Arbor, MI, USA; 5 Department of Obstetrics and Gynecology, University of Michigan, Ann Arbor, MI, USA; 6 Department of Internal Medicine, University of Michigan, Ann Arbor, MI, USA; 7 School of Nursing, University of Michigan, Ann Arbor, MI, USA; 8 Michigan Institute for Clinical & Health Research, University of Michigan, Ann Arbor, MI, USA; 9 Friends of Parkside, Detroit, MI, USA; 10 School of Information, University of Michigan, Ann Arbor, MI, USA; 11 Department of Learning Health Sciences, University of Michigan, Ann Arbor, MI, USA

**Keywords:** Community engagement, equity, virtual community engagement, pandemic, virtual research methods, capacity building

## Abstract

**Introduction:**

The COVID-19 pandemic accelerated a trend for clinical and translational community-engaged research in adapting to an increasingly virtual landscape. This requires a framework for engagement distinct from in-person research and program activities. We reflect on four case studies of community engagement activities that inform a conceptual framework to better integrate the virtual format into community-engaged research reflecting key tenets of health equity and antiracist praxis.

**Methods:**

Four projects were selected by community-engaged research stakeholders for an in-depth review based on how much the virtual transition impacted activities such as planning, recruitment, and data collection for each project. Transitions to virtual engagement were assessed across ten areas in which community engagement has been demonstrated to make a positive impact.

**Results:**

Our analysis suggests a conceptual evaluation framework in which the ten community engagement areas cluster into four interrelated domains: (1) *development, design, and delivery*; (2) *partnership and trust building*; (3) *implementation and change*; and (4) *ethics and equity*.

**Conclusions:**

The domains in this conceptual framework describe critical elements of community engaged research and programs consistent with recommendations for health equity informed meaningful community engagement from the National Academy of Medicine. The conceptual framework and case studies can be used for evaluation and to develop guidelines for clinical and translational researchers utilizing the virtual format in community-engaged research.

## Introduction

The COVID-19 pandemic accelerated the necessity for community-engagement (CE) research activities to be available in online virtual settings, a marked shift from the traditional face-to-face best practices for this type of research. CE research activities are derived from processes that promote inclusive partnerships between researchers and groups of people who affiliate geographically or through an interest in addressing community needs. Partnerships and coalitions that form as part of CE research are designed to mobilize resources and empower stakeholders to build relationships that catalyze changes in policy or practice. In health promotion programs, these activities are central to trust building, acquiring resources and allies, improving communication, and promoting improved health outcomes [1]. Core values of CE include (1) a shared understanding of CE research between investigators and communities; (2) strong community-investigator partnerships; (3) equitable power and responsibility sharing between communities and investigators; (4) inclusion of diverse perspectives and populations; (5) well-defined research aims with shared benefits from research activities among partners; (6) capacity-building opportunities that include continuous communication, transparency, and clarity surrounding ownership and dissemination; and (7) sustained relationships at conclusion [2]. In the USA, the experience of the COVID-19 pandemic as well as protests in support of the Black Lives Matter movement during the summer of 2020 shined a national spotlight on health inequities and systemic racism, renewing focus on the urgency of using meaningful community-engaged research (CEnR) to leverage its unique capacity to address these serious societal challenges and ensure that research reflects the preferences of the community and not a superficial level of engagement in which the community has limited involvement [3]. Meaningful CEnR involves collaborating with communities to foster partnerships in which community members are able to share preferences, provide ad hoc consultation, or engage in shared authority over how research activities take place [4].

To guide researchers and practitioners in CE, several models have been offered that inform best practices. One model, the Spectrum of Public Participation, originally developed by the International Association for Public Participation, is centered on five modes in which communities can participate. These modes form a continuum designed to provide community organizations a framework for determining how the public can engage in democratic decision-making together. The five modes progress from *inform* in which the public is provided balanced information about a community challenge to *consult, involve, and collaborate,* in which public feedback is gathered and analyzed and partnerships are created with the public for decision making. The final mode, *empower*, centers on the return of control and management to the public in place of institutional leaders [5]. Another conceptual model, the Assessing Community Engagement (ACE) Conceptual Model, developed by the National Academy of Medicine’s Leadership Consortium: Collaboration for a Value & Science-Driven Health System, provides a paradigm for how to integrate health equity with the assessment of meaningful CE [4]. Health equity is defined as maximizing individual health through fair and just opportunities to promote health through the removal of obstacles including poverty, racism, discrimination, and lack of access to fair pay, quality education, housing, and health care services [6,7]. The ACE Conceptual Model is centered on four “petals” that reflect the major domains that can be achieved with meaningful CE including (1) strengthened partnerships and alliances; (2) expanded knowledge; (3) improved health and health care programs and policies; and (4) thriving communities. Achieving impact in these domains is designed to build toward the goal of health equity and systems transformation characterized by drivers of health, change in health and healthcare, and social, political, racial, economic, historical, and environmental contexts [4]. As researchers and community partners continue to work toward meaningfully conducting CEnR in the wake of the pandemic, there is an opportunity to identify new ways to uphold the core values and benefits of CE while adapting to virtual formats that have not traditionally been central to CEnR design.

While there have been contributions to the literature related to how the virtual format impacted CE activities and CEnR early in the pandemic [8,9], there is not a standardized framework or set of recommendations on how to effectively utilize the virtual format in CE activities and CEnR in clinical and translational research. In this paper, we reflect on four case studies of CE activities that inform a conceptual framework that can be used to understand how the virtual format can be better integrated into CenR. This framework incorporates the nine impact areas previously identified by the National Institute for Health Research (UK) [10] and the Clinical and Translational Science Awards Consortium (CTSA) Community Engagement Key Function Committee Task Force on the Principles of Community Engagement [1]. These nine impact areas were developed as part of a structured literature review designed to increase awareness of the evidence related to public involvement in health and social care research and are fundamental to the CTSA Task Force’s definition and organizing concepts of CE. We selected these principles as foundations to our conceptual framework to anchor it in the principles that characterize benefits to CEnR. In the conceptual framework presented here, we add a critical tenth impact area – equity – guided by key tenets of antiracism praxis for research in public health as defined by the Public Health Critical Race praxis (PHCR) [11,12]. PHCR stems from critical race theory (CRT) and provides the public health research community with a framework for conducting health equity research retaining fidelity to CRT’s central constructs and expanding beyond reporting on health inequity toward conceptualizing and challenging power structures underlying inequities in communities [11]. This tenth impact area is added to form four interconnected domains – *development, design, and delivery*; *partnership and trust building*; *equity and ethics*; and *implementation and change*.

In this paper, we consider how CE can effectively adapt to virtual formats while continuing to promote its “best practices” in academic and community partnerships [1,10,13]. We present four specific examples (case studies) to examine how the virtual setting both enhanced and posed challenges to CE in these ten core impact areas and provide recommendations for how clinical and translational research can be effectively adapted to the virtual environment, which will likely continue to be an important aspect of CE far beyond the COVID-19 pandemic.

## Materials and Methods

In May 2020, the University of Michigan (U-M) Institute for Healthcare Policy and Innovation (IHPI) formed the Equity and Ethics Community Engagement COVID-19 Interest Group, a group of 25 faculty, community leaders, and program staff who were selected based on decades of experience leading and facilitating CEnR activities. This Interest Group was tasked with the responsibility to identify how the pandemic was impacting community partners and community-engaged activities. Some key areas identified were (1) support for community partners with urgent needs; (2) outreach to community members; (3) building trust and capacity; and (4) developing best practices for using Zoom and virtual collaboration tools. To support CE in the virtual environment, this interest group met to identify existing community engagement research projects and programs. In October and November 2020, a team of U-M IHPI staff and graduate students engaged principal investigators, program managers, and community members leading CEnR activities to answer questions (Table 1) related to the transition to virtual engagement – including planning, recruitment, and data collection adaptations, community partnership impacts and lessons learned, and approaches stakeholders found helpful or not helpful. A convenience sample of four projects was selected for an in-depth review and case analysis consistent with methodology for case selection in qualitative research. We employed a diverse case selection strategy identifying projects that were representative and demonstrative of potential variation across engagement modalities prior to the pandemic (hybrid and in-person) and reflect CE core values, meaningful CEnR, the Spectrum of Public Participation, and the ACE Conceptual Model [14–16].

**Table 1.** Summary of questions for principal investigators, program managers, and community members related to the transition to virtual engagement

Area of focus	Questions
Research activities	• Please provide a brief description of your study or program’s transition to virtual engagement. • What aspect of the research did the virtual transition impact the most (i.e. planning, data collection, etc.)? • What did you do to adjust to conducting virtually? • How did you prepare for conducting research using virtual engagement? • What aspects should remain and what aspects should not remain?
Community participation	• Describe the nature of the existing relationship with the community. • What new relationships developed as a result of the transition to virtual engagement? • How did the research that you conducted virtually intersect with core principles of community-based participatory research (CBPR)? • What are your lessons learned?

The four case studies (Table 2) provide examples of CEnR activities impacted by the transition from either fully in-person or hybrid (virtual and in-person) to fully virtual settings at the onset of the COVID-19 pandemic. These case studies are split into two categories of activity – (1) the *in-person* category is defined as activities that did not incorporate virtual engagement prior to the onset of the pandemic and (2) the *hybrid* category is defined as activities that incorporated both virtual and in-person engagement prior to pandemic’s onset. Table 3 provides more detail related to the impact of the transition to the virtual format.

**Table 2.** Summary of four community engagement case studies

Activity type	Title	Summary of activity
In-person	Young Men’s Health Matters	This research project was designed to identify and sustainably provide healthcare and health education resources to young Black men in Detroit, Michigan.
	Lifecycle of Data	This research project was designed to better understand the Michigan residents’ concerns about how health information should be used, shared and regulated, and influence policy decisions.
	Community Engagement Studio	This program offers opportunities for community members and patients to strategically engage in all stages of clinical and translational research, especially research design and implementation.
Hybrid	DECIDERS	This program developed and evaluated a mechanism to engage communities, particularly minority and underserved communities, in informed deliberations about health research spending priorities. The DECIDERS Steering Committee (SC) originally convened in 2011 with the aim of uniting diverse communities and community partners to increase their voices in priority setting for health research.

**Table 3.** Impact of changing from in-person and hybrid delivery modes prior to COVID-19 pandemic to fully virtual modalities in four community engaged research case studies

Case	Original mode of delivery	Pre-pandemic engagement activities	Adaptations made to accommodate fully virtual modality
Young Men’s Health Matters	In-person	• Community advisory board formed (**meeting in-person**) between Detroit FQHC and University of Michigan School of Nursing • Three community forum meetings held **in-person** in Detroit during 2019 in which the community identified primary concerns • Early 2020 the community advisory board started planning an **in-person summit** in Detroit to address the issues identified in the community forums • **In-person summit** plans included keynote speakers, focus groups, and small group discussions	• Community advisory board transitioned to **meeting virtually** • A **program wide online listserv** was created to facilitate sharing of events, community resources, relevant research articles, and Black men’s health outcomes • In-person summit re-developed as a **virtual event** • Nearly 200 people across the country were able to register for the event and participate because it was **offered virtually**
Lifecycle of Data	In-person	• Six public deliberations were planned **in-person** to examine the public’s preferences on how to improve institutional policies related to precision oncology health information exchange • Two **in-person** deliberations took place in Ann Arbor • Participants limited to those who could travel to the local venue for a **one day** (**8 hour) in-person** deliberation • Meals and venue needed for the **in-person** deliberation	• Remaining four deliberations were **half day (5 hour) virtual meetings** based on community input • Meals and venue were no longer required • Larger pool of potential participants • Barriers related to transportation eliminated • Internet access and technology literacy were newfound barriers mitigated by making all materials available via postal mail and ensuring event and surveys were accessible via smartphone and **conducting Zoom trainings** for participants
Community Engagement Studio	In-person	• Seven i**n-person consultative sessions** with various stakeholders to discuss and evaluate how proposed research studies can be community engagement oriented • Participants recruited locally	• 28 **virtual consultative sessions** with investigators at U-M, local health systems, local health departments, and community-academic partnerships • Participants recruited statewide
DECIDERS	Hybrid	• 18-member Steering Committee met via teleconference, webinar, and **occasional in-person** meetings • CHAT (Choosing All Together) simulation used for health research priority setting had a **virtual** component but required **in-person** deliberations	• Steering Committee transitioned to only meeting **virtually** • Co-PIs started adapting CHAT to be fully **virtual** • While the **virtual transition** allows for more geographically diverse participants, the **virtual format** created **challenges** for achieving the same type of **in-person deliberation** experience

### Young Men’s Health Matters

The *Young Men’s Health Matters* project was originally designed as an in-person study with the goal of identifying and sustainably providing healthcare and health education resources to young Black men in Detroit, Michigan through a community advisory board created in partnership with Detroit Community Health Connection (a federally qualified health center) and the U-M School of Nursing. Three community forums were held in-person in June, August, and October 2019 on both the East and West sides of Detroit to identify community health concerns. Community participants represented local businesses, non-profits, health centers, mental health agencies, literacy programs, faith-based organizations, and youth organizations. The community advisory board originally planned to host a Men’s Health Summit in March 2020 with speakers and group discussions addressing topics related to health and healthcare in urban settings, and field a survey of participants related to these topics. The summit planning was stalled due to the onset of the COVID-19 pandemic, at which point the community decided to move the event to a virtual platform and create a participant and researcher listserv. This virtual two-part event took place in June 2020. Nearly half of all participants were from the Detroit Metro area and the other half came from cities across the country, including San Diego and Washington, D.C. – an aspect of the program made possible by the virtual format.

### Lifecycle of Data

The *Lifecycle of Data* project was originally designed for in-person delivery, consisting of six public deliberations (143 participants) in Michigan to examine the public’s preferences, explore levels of acceptance, and to make recommendations to improve institutional policies and practices on how health information should be used, shared, and regulated in precision oncology. Two of these deliberations took place in Ann Arbor, MI, in-person, prior to the initial COVID-19 outbreak. After a pandemic pause, the remaining four deliberations were carried out virtually with residents from Detroit, Clare County, Grand Rapids, and Northern Michigan.

### Community Engagement Studio (CES)

The *CE Studio* program (CES) is part of the U-M Clinical and Translational Science Award (CTSA) site, the Michigan Institute for Clinical and Health Research (MICHR)’s Community Engagement (CE) Core, in partnership with the MICHR Participant Recruitment Core. The CES program, adapted from the Vanderbilt model, was originally designed to provide in-person only consultative sessions with patients, caregivers, health care providers, community members, and other non-researcher stakeholders to enhance research projects and opportunities for community members and patients to meaningfully engage in all stages of clinical and translational research [17]. Faculty and staff experienced in CEnR (the CE Studio team) facilitate CE Studio sessions with community members and patient stakeholders (known as Community Engagement Studio (CES) experts) to discuss and evaluate proposed research studies including protocols, materials, recruitment, dissemination, etc. The program, launched in August of 2019, was conducted in person. The CES team conducted a total of seven sessions until March 2020. In March 2020, the MICHR CES team shifted the program activities onto Zoom and hosted 28 virtual studios with investigators at U-M, local health systems, local health departments, and community-academic partnerships [17]. A total of 335 CES experts attended these sessions.

### Deliberative Engagement of Communities in Decisions about Research Spending (DECIDERS)

The *DECIDERS* Steering Committee first convened in 2011 with the aim of engaging minority and underserved communities in deliberations about health research spending priorities through a hybrid approach of both in-person and virtual meetings. Subsequent projects have aimed to engage diverse communities and community partners to increase their voice in health policy, particularly spending priorities related to health. Given the statewide nature of the network, since its inception, most monthly membership meetings occurred remotely, with occasional in-person retreats. Community engagement activities using the simulation game “Choosing Healthplans All Together” (CHAT), designed to engage the public in health care priority setting, were facilitated in-person. In March 2020, all program activities transitioned to fully virtual modalities. DECIDERS carried out virtual town hall events in Northern Michigan, a primarily rural community in March 2021, to develop action plans around community health concerns. The program team also developed techniques and visual aids to maximize interactive engagement including the use of Tableau (Salesforce, Seattle, WA) to create maps to illustrate the impact of environmental health problems on the community and an adaptation of CHAT to a fully virtual tool for remote use.

### Evaluation Framework

We examined each of these research projects and programs that transitioned to virtual engagement across nine areas in which CE has been demonstrated to make a positive impact [1,10]. The nine areas include (1) agenda; (2) design and delivery; (3) implementation and change; (4) ethics; (5) the public involved in the project; (6) academic partners; (7) individual research participants; (8) community organizations; and (9) the general public (Table 4). In order to acknowledge issues of fairness, privilege, and discrimination and given CE’s intended impact on social justice, we identified *equity* as a tenth area of focus. This area of focus draws from the Public Health Critical Race praxis [11,12] which articulates a four phased roadmap for guiding researchers from conceptualization to implementation. In identifying equity, we draw on PHCR’s second focus of knowledge production whose purpose is to understand how racialization characterizes a research study. In our conceptual framework, virtual CEnR activities empower communities to articulate whether racialization has informed knowledge related to a project, how virtual tools could facilitate or impede participation, and whether research findings support racial equity. It also integrates the PHCR principle of *voice* [11,12], which prevents imbalances by guiding the use of virtual tools in CEnR activities to enhance CE researchers’ abilities to hear the voices of marginalized populations. This addition also represents a larger movement toward including health equity as a central construct in public health frameworks such as the ten essential services of public health [18].

**Table 4.** Community engagement impact areas [1,6]

Domain	Impact areas	Impact of community engagement
Development, Design, and Delivery	Agenda	May impact choices of topics, research questions, how projects are initiated, and funding decisions.
	Design and delivery	May improve all stages of the research cycle including design, methods, recruitment, data collection, analysis, and dissemination.
Partnership and Trust Building	The public involved in the project	Community members may acquire new knowledge, support, satisfaction, and financial reward. May lay a groundwork of trust and goodwill for future collaborations.
	Academic partners	Academic partners may gain better understanding of the community and have increased opportunities for future collaboration and wider dissemination of findings, which may have positive impacts on career advancement.
	Individual research participants	May improve participant experiences, including improved research processes, access to information and services, and fewer barriers to participation.
	Community organizations	May increase recognition and credibility or community organizations, promote new partnerships with academic researchers and other collaborators, as well as increase knowledge and capacity to benefit their community.
	The general public	May enhance trust and acceptance of the research and promote greater community benefit from research outcomes.
Implementation and Change	Implementation and change	May provide insight on how research findings can enable change, in addition to promoting new partnerships and capacity building.
Ethics and Equity	Ethics	May improve the consent process, identifies ethical pitfalls, and develops processes for resolving ethical issues.
	Equity	Prioritizes values of social justice and fairness to empower partners and participants. Accounting for historic disadvantage and acknowledging systemic racism, may improve processes and outcomes.

### Conceptual Framework

**Fig. 1.**
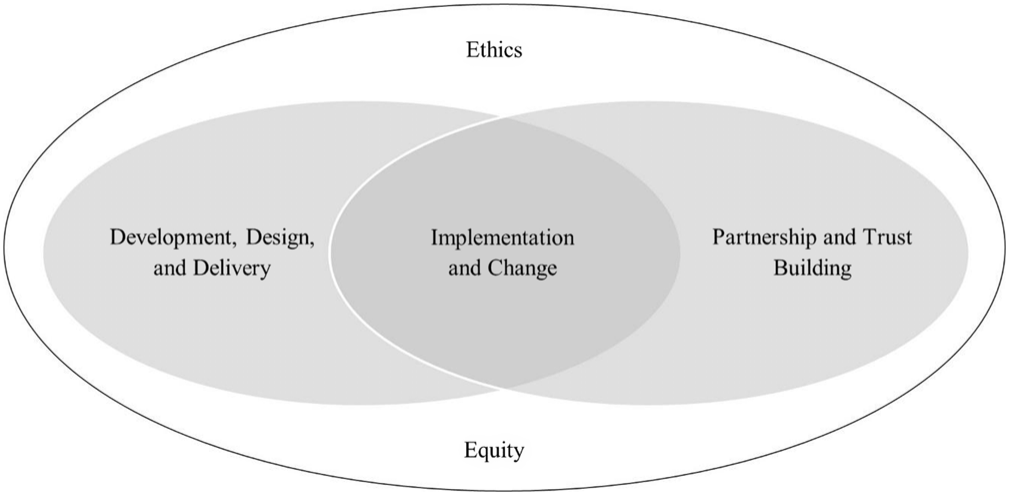
Conceptual framework for virtual community engagement.

The research team initially applied all ten focus areas to each case study to understand the impact of virtual engagement on each of the focus areas. We then used an iterative process to arrive at a consensus around each focus area. The goal of this process was to reduce redundancy, identify themes, and arrive at the most parsimonious domain set. Our conceptual framework resulted from clustering the ten areas into four interrelated domains – (1) *development, design, and delivery* (incorporating areas 1–2); (2) *partnership and trust building* (areas 5–9); (3) *implementation and change* (area 3); and (4) *ethics and equity* (areas 4 and 10). Implementation and change characterize the intersection of the first two domains – bridging *development, design, and delivery* and *partnership and trust building* while *ethics and equity* encompasses all three domains (Fig. 1). The domains in this conceptual framework describe critical elements of virtual community-engaged research.

The first domain, *development, design, and delivery*, incorporates the CE impact areas including *agenda* and *design and delivery*. The impact of CE on *agenda* relates to choices of topics, research questions, and how projects are initiated and funded. The impact on *design and delivery* focuses on how all stages of the research cycle including methods, recruitment, data collection, analysis, and dissemination are enhanced by community participation. The second domain, *partnership and trust building*, incorporates the CE impact areas including the *public involved in the project*, *academic partners*, *individual research participants*, *community organizations*, and *the general public*. These areas encompass multiple levels of CE from the individual participant, community stakeholders, and organizations, to the general public. These impact areas focus on improving the community’s research experience, lowering barriers to participation, improving community access to knowledge and resources, and promoting greater trust through collaborative relationships and capacity building that benefit communities.

The third domain, *implementation and change*, focuses on how research findings can enable change and promote new partnerships and capacity building and how this facilitates *development, design, and delivery* and *partnership and trust building*. Finally, the fourth domain, *ethics and equity*, incorporates CE impact areas that permeate and are at the heart of CE and CEnR. Although the familiar ethical principles of research apply to CE (whether in-person or virtual), CE also includes a broader set of principles and issues outside of the traditional focus on the autonomy of research subjects, often limited to the of informed consent [19,20]. Ethics and equity in CE expand on the foundational Belmont principles of justice, respect for persons, beneficence, and non-maleficence to include social justice and fairness in ways that prioritize the empowerment of partners and participants and account for historical disadvantage and dismantling of systemic racism. CE researchers and practitioners have long recognized that the individualized focus of research ethics is insufficient to address equity because it does not account for the social, economic, and political structures that perpetuate racism [21].

## Results

We have organized the results according to the different domains described above and discuss the principles in the impact areas as they relate to each interrelated domain in the conceptual framework.

### Development, Design, and Delivery

For many CEnR projects, suspending in-person activities due to the COVID-19 pandemic was a potential barrier to community engaged agenda setting. In the case of Young Men’s Health Matters, when the community advisory board pivoted to meeting virtually, it was able to invite keynote speakers who would not have been able to participate in person and to provide additional education that catalyzed subsequent collaborations. The virtual platform also permitted more inclusive focus groups with participants from across the USA providing a more inclusive opportunity to participate in CE and survey research that led to tangible findings for the health and wellness of urban communities.

In the case of DECIDERS, the already established hybrid mode of delivery enabled the program team to adapt these virtual approaches to fully replace in-person meetings, allowing agenda and priority setting to continue. Program partners also investigated additional techniques to maximize interactive engagement. Tableau was used to create maps that could illustrate various environmental health problems and their relationship to social vulnerability. The DECIDERS team also initiated the process of redeveloping a fully virtual version of the CHAT simulation used for informed deliberation about limited resources to allow for future uses of CHAT without face-to-face interaction.

The virtual pivot enabled the CES team to continue to engage communities in informing how research is carried out and expanded the reach of the program beyond Southeast Michigan as CES experts located on the west, northwest, northeast, and Upper Peninsula of Michigan were now able to participate easily in CES events. Similar to the CES program expansion, recruitment strategies for Lifecycle of Data also changed as the pool of potential participants was expanded and community partners were able to recruit beyond the local area.

For academic partners to leverage the virtual format, Zoom training of study teams and facilitators was necessary along with the development of virtual recruitment techniques to facilitate a structured virtual pivot. This was especially important to the Lifecycle of Data research project which conducted Zoom trainings for its study team, requiring additional time to make a smooth transition. It was also important to evaluate the appropriateness of switching to virtual formats, given the impact of the pandemic, i.e., the context in which a virtual format was necessary. As part of the CES program, CES experts were provided with virtual platform training, allowing them to be more involved in program activities – especially early in the pandemic when technology literacy was more nascent. Since the DECIDERS steering committee had already been meeting virtually prior to the pandemic, they were able to continue their practice of meeting virtually every month and offer mentorship and support related to virtual CE methods to other CEnR projects.

### Partnership and Trust Building

The virtual format was especially helpful in promoting greater public involvement in CEnR activities. For example, the CES program was able to run more easily and empower academic partners to be better informed of real-time community concerns building trust among the CES experts. The virtual format also empowered participants to engage more easily with CEnR activities allowing partnerships with community organizations to continue to grow. In the Life Cycle of Data project, participants expressed that they felt the virtual format allowed their concerns to be heard by the research team and that virtual conferencing tools provided greater opportunities to build trust as they engaged in the research activities. In all four case studies, community partnerships that grew as a result of the virtual transition allowed for easier dissemination of outcomes to both participants and the public, improving the public’s receptivity to the findings and thereby strengthening partnerships through trust building.

### Implementation and Change

Opportunities to promote capacity building and foster change among communities were another outcome of the virtual transition. In Young Men’s Health Matters, the research team was able to continue meeting with the community advisory board and sustain partnerships in the community through the implementation of the community listserv to disseminate findings and future projects. This further enhanced the design and delivery of the intervention and strengthened the trust building taking place as part of the study.

DECIDERS completed a participatory, longitudinal evaluation of the partnership using virtual techniques that confirmed strong relationships and set goals for future work, including an emphasis on building the capacity for partners to work with multiple projects and researchers, and emphasizing the priority of research that generated demonstrable community health impact.

### Ethics and Equity

CEnR recognizes the centrality of ethics and equity to the development, implementation, partnership, and trust building of CE activities. Increased adoption of virtual modalities in the wake of the pandemic heightened three core tenets of ethics and equity in CEnR. First was respect for the needs and priorities of community and academic partners. All of the activities described here stopped ongoing projects as part of a larger pause in all research activities at U-M, and CEnR teams needed to assess and understand the impact of the pandemic on both the personal and professional activities of research staff and community members. As a result of this reflective process, stronger and ultimately more equitable partnerships were built through increased trust that supported the virtual work that would follow. In the Lifecycle of Data project, the shift to virtual formats allowed for geographic catchment areas that were larger than anticipated. For those who had in-person focus groups as a point of comparison (n = 35), most found group discussion the same as or easier using Zoom (84.7%). Second, as it became clear that virtual modalities were necessary, CEnR teams needed to consider the implications for inclusion and capacity building. Across the case studies, research teams worked to increase virtual access for community members through training of partners and community members creating a more inclusive and equitable partnership in which barriers to participation were removed. The need to reassess recruitment and communications strategies also allowed for more equitable CE. For example, monthly DECIDERS steering committee meetings began with questions about community needs as the geographically and culturally diverse communities of DECIDERS had varied experiences, different trajectories of surges and needs, and could share information, strategies, and concrete assistance with each other. Third, the virtual format provided opportunities for staff and community members to become more proficient about the implications of consent. In the CE Studio case, CES experts participating in CES program activities were able to provide input on how to appropriately work with participants to ensure robust processes and the ethical concerns of the communities they represent. As a result, new research areas emerged such as the Life Cycle of Data team’s expanded research related to examining trust and the impact of COVID-19 on Detroit public housing residents [22].

## Discussion

Community-engaged research has been reluctant to adopt a virtual model because of the distinctive features of established rapport, relationships, and investment in communities that have traditionally characterized in-person activities [23–26] While virtual engagement is not an in-person replacement, it is a new modality that can be leveraged to expand access, increase equity, improve trust building, and enhance the delivery and implementation of CEnR. Therefore, engagement activities that take place virtually need to reflect a set of best practices that allow for equitably involving community members, organizational representatives, and researchers throughout the process [4,13]. Each of the four domains of the conceptual model presented here identified both benefits and challenges when shifting to a virtual format that can inform best practices.

### Benefits of Shifting to Virtual Formats

Virtual CE allowed researchers and community advocates to expand the reach of their activities especially enhancing recruitment. In each case study, research activities grew to include participants across the State and the USA, subsequently making resources and educational opportunities accessible to a broader audience. Once community partners had systems in place for virtual activities, there was a marked shift in first-time participation as virtual formats lowered the burden of participation to attract individuals who had not previously volunteered to participate, removing impediments to both recruitment and participation. For example, among virtual participants in Lifecycle of Health Data, 41.7% had not previously participated in an in-person focus group.

Virtual community engaged research events provided participants an opportunity to communicate the barriers and stressors they were facing more easily. In addition, whereas in-person gatherings only provided those who were able to attend the opportunity to share their perspectives, the Zoom format provided a more representative group of participants from across the State the ability to use various modalities such as video, chat, and screen sharing to share more about the barriers they were facing. These structured opportunities to share perspectives with others and to create new connections were a newfound benefit of this shift to virtual formats, allowing for both more equitable representation of thoughts and ideas and additional opportunities for partnership and trust building. The virtual format also provided a more seamless way for researchers to follow-up with participants. Study results were more easily disseminated to participants via email and more participants returned for virtual presentations to review findings compared to the in-person presentations that took place prior to the pandemic. The virtual format also enabled research staff to stay in touch with participants and notify them of other research opportunities.

### Challenges in Shifting to Virtual Formats

While the virtual format facilitated more inclusive participation, it also presented challenges to stakeholders. Whereas in-person events did not require specific skills or training for participation, the transition to virtual platforms required significant training for both research staff and participants. Research teams needed to develop an organized and clearly documented process for assessing participant comfort or familiarity with virtual platforms to validate participation ability before the day of virtual events, creating a barrier for some to engage in research activities. For those unfamiliar with virtual platforms, a training plan and identification of a core training team was necessary to ensure a positive experience for participants supporting the fidelity of the research model. It was also necessary to consider internet access and bandwidth in determining a path to successful participation. In some cases, potential participants did not have the necessary computing infrastructure (microphone, camera, stable internet) to participate in virtual community engaged research and needed to adapt their participation further through mobile device usage. While local community partners were able to provide support and the computing infrastructure in some cases, partnership with local community agencies was not always feasible or available. This challenge exacerbated some aspects of inequitable rates of participation as those who did not have reliable internet access or adequate training were systematically excluded from participation even though the virtual format alleviated other commonly encountered barriers to in-person participation such as the need to secure childcare or transportation.

Similarly, CE tools that were adapted to an entirely virtual version revealed critical technological barriers as creating a quality fully virtual version of these tools required integrating the need for participants to have high-quality internet access, a laptop or desktop computer, training on Zoom, and the ability to convene via Zoom and in a web browser. The need to accommodate these attributes impeded research teams’ abilities to successfully achieve the transition to a virtual offering of tools that were previously successful in the in-person setting.

### Future Applications of Our Framework to CEnR Activities

This framework offers a way of examining the experience of CEnR projects. However, the categories are fluid and not all activities will align exclusively to a domain under all circumstances. One example of potential misalignment in our analysis was related to Zoom training of participants and study team members. Zoom training provided to partner organizations by the study team had trust building and partnership as an outcome; however, it could equally be seen as belonging in the *development, design, and delivery* domain as a process employed by the research team to facilitate delivery of a CEnR project. Another limitation of our conceptual framework is our use of a convenience sample to identify the four projects selected for in-depth review. Future research might utilize a different sampling technique to be able to identify more projects for in-depth review in order to include both typical cases as well as extreme, deviant, or influential cases [14]. Nonetheless, our conceptual framework provides a way to organize research activities and structure how researchers might approach using the virtual format in community engaged research activities to ensure that they consider the different dimensions of using the virtual format in order to meet the same goals as in-person and hybrid projects.

### Implications for Practice and Research

Consistent with the recommendations for meaningful CE as part of the National Academy of Medicine’s ACE Conceptual Model, virtual CE must actively uphold the value of equity. Revised guidelines and best practices should be developed as explicitly anti-racist [12,27], irrespective of whether engagement activities are conducted in virtual or in-person settings. As our case study examples suggest, the type of engagement that occurs, and what type of modality is used have implications for inclusion, access, and participation.

While training materials and resources related to CE reflect how to utilize CE with virtual communities built on social media, they do not include best practices on how to use the virtual format to effectively facilitate engagement with communities in research activities. The conceptual framework and case studies presented in this paper provide foundational lessons that can be used to revise these resources so that clinical and translational CEnR – utilizing the virtual format – can take advantage of its benefits and address the challenges that it imposes.
